# In vivo magnetic resonance imaging of the effects of photodynamic therapy.

**DOI:** 10.1038/bjc.1989.244

**Published:** 1989-08

**Authors:** N. J. Dodd, J. V. Moore, D. G. Poppitt, B. Wood

**Affiliations:** Paterson Institute for Cancer Research, Christie Hospital and Holt Radium Institute, Manchester, UK.

## Abstract

**Images:**


*Br. J. Cancer* (1989), **60**, 164–167 © The Macmillan Press Ltd., 1989

### *In vivo* magnetic resonance imaging of the effects of photodynamic therapy

N.J.F. Dodd, J.V. Moore, D.G. Poppitt & B. Wood[1]

*Paterson Institute for Cancer Research, Christie Hospital and Holt Radium Institute, Manchester M20 9BX and [1]Biomedical NMR Unit, University of Manchester Medical School, Manchester M13 9PT, UK.*

**Summary** Nuclear magnetic resonance (NMR) proton imaging and measurements of the parameters $T_1$ and $T_2$, have been carried out *in vivo* on the murine mammary tumour T50/80. Tumours had been treated 24 h previously by photodynamic therapy (PDT, using haematoporphyrin derivative and 630 nm laser light). Proton images clearly demarcated a high signal-intensity region on the side of the tumour closest to the incident light beam, while the parts of the tumour more remote from the beam resembled the images from untreated controls. Both $T_1$ and $T_2$ values were raised in the high-intensity region. This high-intensity region was shown to correspond to PDT-induced histological necrosis, the low-intensity region to histologically intact tumour. Linear regression analysis of the relationship of depth of necrosis measured histologically and 'depth of necrosis' measured from the NMR images, yielded a slope of 0.93 ($r^2=0.95$).

There is increasing experimental and clinical interest in photodynamic therapy (PDT), in which the combination of visible light and photosensitising drugs produces locally cytotoxic chemical moieties. A principal mode of action of PDT *in vivo* is thought to be acute injury to the vasculature of both tumours (e.g. Henderson *et al.*, 1985) and normal tissues (e.g. Berenbaum *et al.*, 1986; Benstead & Moore, 1988). In tumours, vascular collapse leads to prompt and massive secondary ischaemic necrosis of the malignant cells. This classical form of cell death is characterised by failure of large numbers of contiguous cells to maintain homoeostatic regulation of fluid volume, together with early cessation of mitochondrial energy metabolism (Trump & Arstila, 1975). This contrasts with the form of cell death ('apoptosis'; reviewed by Kerr *et al.*, 1987) that occurs after, e.g., ionising irradiation, where cells die as individuals scattered among groups of apparently intact, metabolising cells. It appeared to us that the former type of cell death might offer opportunities for proton imaging of acute injury to tumours by PDT. In the current stage of development of PDT, some form of early monitoring of treatment would be of great value, as the relationships of delivered PDT 'dose' and biological effect are not simple. Light 'dose' is commonly defined in terms of fluence at the tumour surface, while drug 'dose' is almost universally the amount injected into the animal or patient; the actual quantities of each within tumours are likely to vary between individuals because of biological heterogeneity, leading to a variable therapeutic response. Threshold doses of both light and drug are required to achieve cell killing. We report here our first results on the use of NMR in monitoring the effects of PDT on an experimental mammary tumour.

### Materials and methods

### *Mice*

Nine- to 10-week-old male mice of the B6D2$F_1$ strain (the $F_1$ hybrid of the cross of sib-mated lines C57B16 x DBA2; Paterson Institute strains) were used. Mice were housed under a 12 h dark (18.00–06.00 h), 12 h light regimen except where otherwise noted, and were provided with food and water ad libitum.

### *Tumour*

Third to 6th passage generations of the mammary carcinoma T50/80, syngeneic in B6D2$F_1$ mice, were used. Tumours were implanted as a brei in the subcutaneous tissues of the abdominal flank, and used experimentally when they reached an average depth, normal to the body wall, of 9 mm (average length of 1.5 cm).

Received 21 November 1988, and in revised form, 17 March 1989.

### *Photosensitising drug*

Haematoporphyrin derivative (HPD; Paisley Biochemicals, Paisley, Scotland) was obtained and used as a 5 mg ml$^{-1}$ solution in 0.9% saline. HPD was injected intraperitoneally at a well-tolerated dose of 40 mg kg$^{-1}$ and the animals were kept in the dark for 24 h and then treated by laser light. The dose of drug, which falls within the reciprocity region for this drug-laser-tumour- effect combination, was selected in order to minimise the length of laser exposure required to achieve the biological effect.

### *Laser irradiation*

A 10 W copper vapour laser (Oxford Lasers, Oxford) was used to pump a dye laser that generated 630 nm red light. This was focused into a 1 mm diameter quartz fibre. The diverging beam from this fibre was directed vertically downward to produce a circular field of 3.5 cm diameter at a distance equivalent to that at which the centre of the tumour would lie. The laser beam was not completely 'flat' normal to the longitudinal axis, accordingly a relatively large beam diameter was chosen in order to minimise inhomogeneities of fluence across the approximately 1.5 cm length of these tumours. The power density of light at this distance was adjusted to 100 mW cm$^{-2}$, as measured by a thermopile. The light doses given were expressed in terms of fluence at this distance, in J cm$^{-2}$. Unanaesthetised mice were placed prone in a jig that protected the whole body other than the tumour from the laser light. Single doses of 87.5–125 J cm$^{-2}$ were given. The vertical axis of the beam was marked on the skin overlying the tumour and the mice were then housed under subdued light for 24 h.

### *NMR*

Proton images of the tumours were obtained using a 'Biospec' system (Bruker/Oxford Research Systems, Coventry), with a 4.7 T, 15 cm horizontal bore, magnet. Animals were anaesthetised during imaging by an i.p. inection of 0.1 ml of a 50 mg ml$^{-1}$ solution of 'Ketalar' (Parke-Davis, Pontypool) and positioned in the same perspex holder that had been used for their irradiation by light. A single turn surface coil (Ackerman *et al.*, 1980) of 3 cm diamater, was placed just above the tumour (Figure 1). Images were acquired in the plane of the coil, using a spin

**Figure 1** Schematic of the imaging set-up (components not to scale). The longitudinal axis of prior laser illumination was in the plane of the paper; the tumour which lay normal to the flank, had been illuminated on its upper aspect; and the RF coil overlay the tumour, again in the plane of the paper.

echo pulse sequence. A 90° 'hard' pulse of between 15 and 40 $\mu$s duration was used, depending on the position of the slice of interest relative to the plane of the coil. A frequency-selective refocusing pulse of 6 ms duration was used in a slice selection gradient of $22\,mT\,m^{-1}$, to give a slice of 1 mm thickness. This particular combination of pulses was used as, in our hands, it avoided rapid changes in magnetic field which can lead to artefacts in imaging. Repetition time (TR) was 1.55 s and echo delay time (TE) was 52 ms. The overall imaging time was 6 min 36 s for a 256 × 256 image, with in-plane resolution of 120 × 120 $\mu$m, for the 3.1 cm field of view. To obtain $T_1$ and $T_2$ times, phase encoding was reduced to 128 steps. For measurement of $T_1$, TE was 52 ms and TR was varied from 0.25 to 5.05 s in 4–6 steps, while for $T_2$, TE was varied from 52 to 142 ms and TR was 5.00 s. Values for $T_1$ and $T_2$ were calculated from:

$$S_{TR} = S_0(1 - e^{-TR/T_1}), \text{ and } S_{TE} = S_0 e^{-TE/T_2},$$

where $S$ = image intensity.

*Histology*

Immediately after NMR imaging, the mice were killed painlessly by anaesthesia. The animals were anaesthetised while still within the jigs in which they had been imaged, in order to maintain the spatial relationships of the planes of laser irradiation, imaging and histology. The tumour was then partially bisected in the marked plane of irradiation, the mouse removed from the jig, and the tumour dissected free and fully bisected. The tumour was then fixed for histology, using Bouin's fluid to prevent shrinkage. Three-$\mu$m thick sections were made from the cut, equatorial faces of the tumour halves and then stained with Haematoxylin and Eosin.

### Results

*NMR*

Proton images of T50/80 tumours that had received drug alone or light alone were indistinguishable from those of untreated control tumours, and were as shown in Figure 2. The images just resolved the normal skin overlying the tumour; the dark region between skin and tumour may be the fibrous capsule that surrounds this neoplasm. Within the tumour mass, the characteristically stippled appearance of T50/80 images is believed to reflect the presence of numerous discrete foci of 'old' coagulative necrosis (Figure 3c). NMR images acquired 24 h after PDT show clearly the effects of therapy (Figure 3a). The peripheral bright area represents the prompt oedema and inflammatory infiltration of the dermis of the skin that is a common feature of external treatments by PDT. On the side of the tumour closest to the incident light beam, an area with high average signal intensity could be distinguished from the image of the tumour regions more remote from the beam (the latter more closely resembling the images obtained from untreated controls; cf Figures 2 and 3a).

**Figure 2** NMR image of untreated T50/80 tumour. The thin overlying skin is just resolved around the bottom right-hand quadrant. Magnification ×4.6.

Average values for the relaxation times $T_1$ and $T_2$ were measured for areas of 1,200 × 1,200 $\mu$m within (a) the oedematous dermis, (b) the high signal-intensity region of the tumour nearest the light beam, (c) the lower-intensity region remote from the beam. Two sites were sampled within each region of each tumour and the data pooled. Values for oedematous skin were significantly ($P<0.05$) higher than for the tumour sites, with respect to both $T_1$ and $T_2$ measured at 200 MHz (Table I). In turn, values for the high-intensity region of the tumour were significantly ($P<0.05$) higher than those for the lower intensity region. Although statistically significant, the differences in $T_1$ and $T_2$ between the two regions of the tumour are insufficient of themselves to account for the differences in the image intensities. For image intensities given by: $S = S_0(1 - e^{-TR/T_1})(e^{-TE/T_2})$, a % contrast between necrotic and viable zones could be calculated by: $100 \times (S_n - S_v)/S_v$. Adopting the values for $T_1$ and $T_2$ given in Table I, then for a TE of 52 ms and TR of 1.552 s the predicted % contrast is 15%, assuming equal proton densities. However, the *observed* contrasts in image intensity in the different tumours ranged from 23 to 44%.

*Comparison of histological sections and NMR images*

The object was to compare the appearance of an NMR image with that of a section through the tumour with which that image corresponded spatially. Accordingly, a uniform method of analysis was adopted for both. Each coded tumour section was scanned at right angles to the incident light beam, at intervals of 0.4 mm. For each position, and in a plane corresponding to the vertical axis of the light beam, measurement was made of the depth of tumour tissue adjudged to have been wholly destroyed by PDT ('necrotic' zone; Figure 3b). This was recognised as containing only congested blood vessels and tumour cells with hyperchromatic, pyknotic or karyorrhectic nuclei, i.e. with no histologically intact cells (defined as those with rounded nuclei containing homogeneous heterochromatin). The inner edge of the necrotic zone was defined as the depth at which the first histologically intact cell was encountered in scanning the plane (below which was the 'viable' zone; Figure 3c). Twenty-five to 35 measurements were made per tumour. Polaroid photographs of the NMR images were analysed similarly. In this case, the 'necrotic' zone was defined as the region of high signal intensity bounded by the tumour edge nearest the incident light beam and extending a variable distance into the tumour mass (Figure 3a).

Figure 3 **a**, NMR image of T50/80 tumour treated 24 h previously by HPD plus $112.5\,J\,cm^{-2}$ of laser light, whose beam direction was vertically downward in the plane of the image. Shown in the image are oedematous dermal skin (D), the PDT-damaged 'necrotic' zone (N) of the tumour, and the deeper 'viable' zone (V). Magnification ×3.4. **b**, Light micrograph of the histology of the tumour zone destroyed by PDT (corresponding spatially to image region 'N' in Figure 3a). The junction of the tumour with its fibrotic capsule is shown (J). All tumour cells are pyknotic (P) and the blood vessels congested (B). Haematoxylin and Eosin staining. Magnification ×85. **c**, Light micrograph of the histology of the tumour zone spared in the PDT treatment (corresponding spatially to image region 'V' in Figure 3a). Shown are intact blood vessels (B), histologically intact tumour cells (T), and 'old' coagulative necrosis (C). The histology of untreated tumours is identical to this illustration. H and E staining. Magnification ×85.

**Table I** *In vivo* NMR relaxation times of tissues, in mice treated 24 h previously by PDT

| *Tissue* | $T_1(s)$ | $T_2(ms)$ |
|---|---|---|
| Oedematous dermis | $2.10 \pm 0.10^a$ | $111 \pm 16$ |
| 'Necrotic' tumour | $1.58 \pm 0.10$ | $52 \pm 7$ |
| 'Viable' tumour | $1.31 \pm 0.11$ | $41 \pm 2$ |

[a]Errors as 1 s.d. for inter-animal variation ($n=4$). Measured at 200 MHz.

The mean depth of necrosis in tumour sections rose from 2.75 to 4.5 mm as the incident light dose was increased from 87.5 to $125\,J\,cm^{-2}$. However, for a given dose there was a large variation in depth of histological necrosis between individual tumours (Figure 4a), and the differences between means for increasing doses were statistically insignificant. Thus, dose was a very poor predictor of PDT effect (using, arbitrarily, a linear regression fit, $r^2=0.055$). In contrast, a plot of depth of histological necrosis as a function of depth of NMR 'necrosis' could be described by the equation: $y=0.13+0.93x$, $r^2=0.95$ (Figure 4b).

### Discussion

For the particular combinations used here of drug dose, drug-light interval, and power density of external illumination, there was a very wide variation in biological effect for a given applied 'dose' of PDT. In an experimental system, it is possible to optimise the different parameters to reduce some of this variation; for example, since carrying out these initial experiments, we have used extraction methods to show that the amount of HPD that remains at 24 h is widely variable between tumours, but much less so at 6 h (J.V. Moore, to be published separately). In patients, such optimisation procedures are more difficult or even impossible, so that a non-

Figure 4 **a**, Depth of histological necrosis in T50/80 as a function of light fluence on the tumour. Circles are mean values for individual mice ±2 s.e. (for 25–35 measurements per tumour; intra-tumour variation). Where errors are not shown, they are smaller than the symbol. Squares are mean depth of necrosis for a given fluence ±2 s.e. (for 3–5 mice; inter-tumour variation). **b**, Depth of histological necrosis in T50/80 as a function of depth of the high-signal-intensity zone in the corresponding NMR image. Mean values ±2 s.e. (for 25–35 measurements per tumour). Line is the linear least-squares fit to the data.

invasive method such as NMR for measuring the effect of a treatment should be of clinical relevance.

Recently, $^{31}P$ NMR spectroscopy has been used to detect the early metabolic responses of tumours *in vivo* to PDT (e.g. Ceckler *et al*., 1986; Naruse *et al*., 1986; Chopp *et al*., 1987). There is a decrease in ATP and increase in inorganic phosphate within 1 h of treatment and continuing for several hours thereafter. With sub-curative treatments, the tumour ATP levels may recover to, or even exceed, that of controls. Thus $^{31}P$ NMR spectroscopy may be a useful method for monitoring effects of PDT, but one that requires repeated measurements and, as presently applied, one that gives information only about the average metabolic state of the tumour (and requires care, particularly with small tumours, to exclude 'contamination' of results by underlying muscle). Our results suggest that proton NMR imaging may provide precise information on the extent of histological damage resulting from a PDT treatment and by inference, on whether further treatment would appear to be required. However, these conclusions rest on a number of assumptions:

(a) That the images do indeed distinguish lethally damaged regions of tumours from 'viable' areas. There is a well-established consensus that cells recognised as severely pyknotic or karyorrhectic (Figure 3b) are reproductively dead (although it does not follow that cells that are histologically intact are necessarily reproductively viable). For the mammary tumours examined here, the correlation between histologically defined areas of tumour destruction and NMR images, was good. Inspection of the two lower clusters of points in Figure 4b suggests that for a given depth of NMR 'necrosis', the histological depth of necrosis might be greater or less by up to 1 mm.

(b) That 24 h is an appropriate time for comparison of image and histology. Full time-course experiments are currently under way but it is known that the expression of necrosis in tumour cells as a result of hypoxic or ischaemic hypoxia occupies approximately 4–11 h (data from various authors, summarised by Moore, 1987). One might therefore expect that by 24 h after PDT, the great majority of dead cells would have expressed necrosis, while recovery processes would not yet have occurred to a major extent (e.g. Star *et al*., 1986; Kaye & Morstyn, 1987). As regards the NMR images, we have noted that at 24 h not only were $T_1$ and $T_2$ values increased in the 'necrotic' zone, but there was an additional image contrast relative to the 'viable' zone. Although the $T_1$ and $T_2$ values may be subject to revision on more detailed measurement (e.g. the assumption of a single exponential decay may be inappropriate), it is likely that this high image contrast represents in part an increase in the water content of the necrotic zone. It is characteristic of PDT injury to tumour and normal tissue that oedema develops promptly within the irradiated region, some 3–6 h after illumination (e.g. Berenbaum *et al*., 1986; Kaye & Morstyn, 1987) and may peak at 1 or 2 days before resolving (e.g. Moore *et al*., 1986; Star *et al*., 1986). The choice of the same time, 24 h, to measure both NMR parameters and histology, seems not inappropriate.

(c) That measurement of depth of necrosis at early times is relevant to therapeutic outcome. Brasseur *et al*. (1987) found that the presence or absence of a strong necrotic reaction 3 days after PDT using a variety of drugs, predicted reasonably well the relative efficacy of the drugs in causing local control of the EMT6 mammary tumour. More directly relevant to the present experiments, Kinsey *et al*. (1983) demonstrated for a murine mammary tumour that the increasing radius of the necrotic zone, measured histologically 2 days after interstitial laser illumination at progressively higher power densities, corresponded with an increasing delay in regrowth of similarly treated tumours. Using non-invasive NMR imaging, it will be possible to test definitively, for individual animals, the precise relationships of early response and therapeutic outcome. Of particular interest is that very preliminary results suggest that it may be possible to obtain similar images using a low-field strength clinical NMR machine (in our case, a 0.26 T, 11 MHz, Picker instrument).

N.J.F.D., J.V.M. and D.G.P. are supported by the Cancer Research Campaign. The Biomedical NMR Unit is supported by the SERC.

### References

ACKERMAN, J.J.H., GROVE, T.H., WONG, G.G., GADIAN, D.G. & RADDA, G.K. (1980). Mapping of metabolites in whole animals by $^{31}P$ NMR using surface coils. *Nature*, **283**, 167.

BENSTEAD, K. & MOORE, J.V. (1988). Vascular function and the probability of skin necrosis after photodynamic therapy: an experimental study. *Br. J. Cancer*, **57**, 451.

BERENBAUM, M.C., HALL, G.W. & HOYES, A.D. (1986). Cerebral photosensitisation by haematoporphyrin derivative. Evidence for an endothelial site of action. *Br. J. Cancer*, **53**, 81.

BRASSEUR, N., ALI, H., LANGLOIS, R., WAGNER, J.R., ROUSSEAU, J. & VAN LIER, J.E. (1987). Biologic activities of phthalocyanines. V. Photodynamic therapy of EMT-6 mammary tumours in mice with sulphonated phthalocyanines. *Photochem. Photobiol*., **45**, 581.

CECKLER, T.L., BRYANT, R.G., PENNEY, D.P., GIBSON, S.L. & HILF, R. (1986). $^{31}P$-NMR spectroscopy demonstrates decreased ATP levels *in vivo* as an early response to photodynamic therapy. *Biochem. Biophys. Res. Commun*., **140**, 273.

CHOPP, M., FARMER, H., HETZEL, F. & SCHAPP, A.P. (1987). *In vivo* $^{31}P$-NMR spectroscopy of mammary carcinoma subjected to subcurative photodynamic therapy. *Photochem. Photobiol*., **46**, 819.

HENDERSON, B., WALDOW, S.M., MANG, T.S., POTTER, W.R., MALONE, P.B. & DOUGHERTY, T.J. (1985). Tumour destruction and kinetics of tumour cell death in two experimental mouse tumours following photodynamic therapy. *Cancer Res*., **45**, 572.

KAYE, A.H. & MORSTYN, G. (1987). Photoradiation therapy causing selective tumour kill in a rat glioma model. *Neurosurgery*, **20**, 408.

KERR, J.F.R., SEARLE, J., HARMON, B.V. & BISHOP, C.J. (1987). Apoptosis. In *Perspectives on Mammalian Cell Death*, Potten, C.S. (ed.) p. 93. Oxford University Press: Oxford.

KINSEY, J.H., CORTESE, D.A. & NEEL, H.B. (1983). Thermal considerations in murine tumour killing using haematoporphyrin derivative phototherapy. *Cancer Res*., **43**, 1562.

MOORE, J.V. (1987). Death of cells and necrosis of tumours. In *Perspectives on Mammalian Cell Death*, Potten, C.S. (ed.) p. 295. Oxford University Press: Oxford.

MOORE, J.V., KEENE, J.P. & LAND, E.J. (1986). Dose–response relationships for photodynamic injury to murine skin. *Br. J. Radiol*., **59**, 257.

NARUSE, S., HORIKAWA, Y., TANAKA, C. and 4 others (1986). Evaluation of the effects of photoradiation therapy on brain tumours with *in vivo* $^{31}P$ MR spectroscopy. *Radiology*, **160**, 827.

STAR, W.M., MARIJNISSEN, H.P.A., VAN DEN BERG-BLOK, A.E., VERSTEEG, J.A.C., FRANKEN, K.A.P. & REINHOLD, H.S. (1986). Destruction of rat mammary tumour and normal tissue microcirculation by haematoporphyrin derivative photoradiation observed *in vivo* in sandwich observation chambers. *Cancer Res*., **46**, 2532.

TRUMP, B.F. & ARSTILA, A.U. (1975). Cellular reaction to injury. In *Principles of Pathobiology*, Lavin, M.F. & Hill, R.B. (ed.) p. 9. Oxford University Press: Oxford.


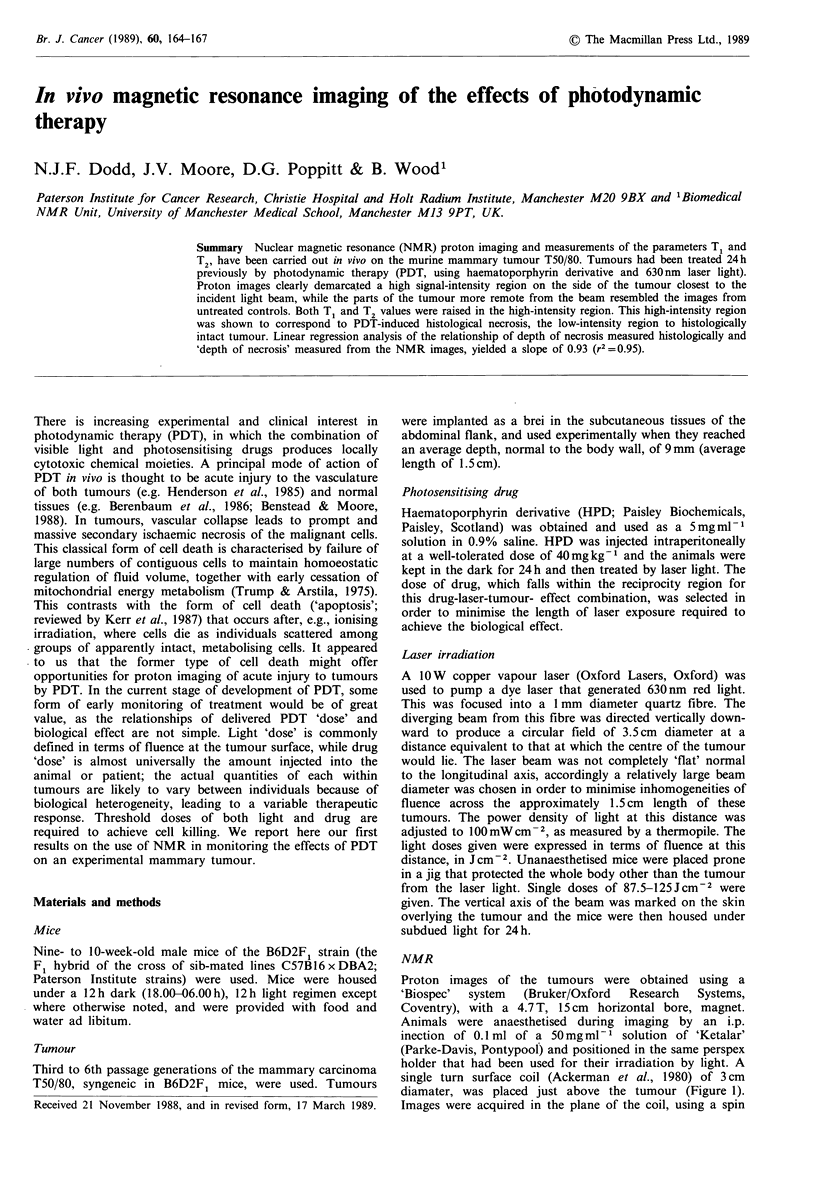

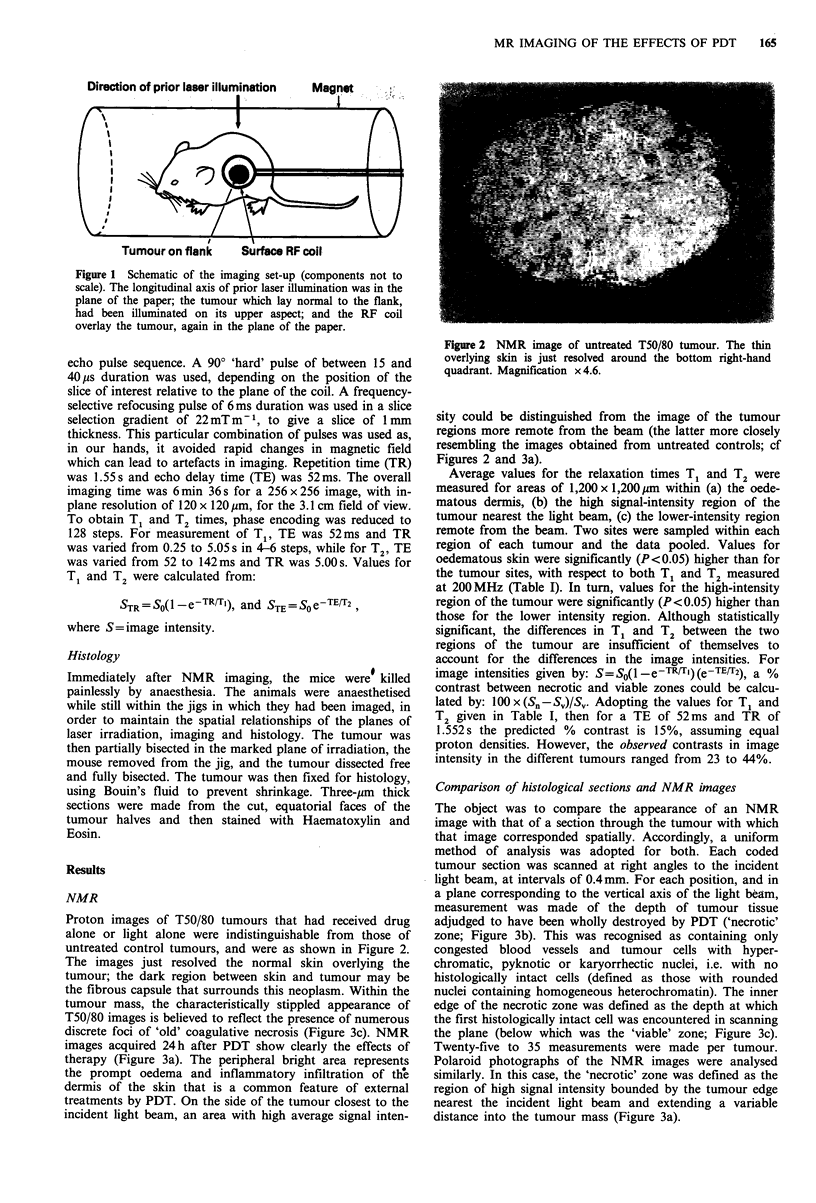

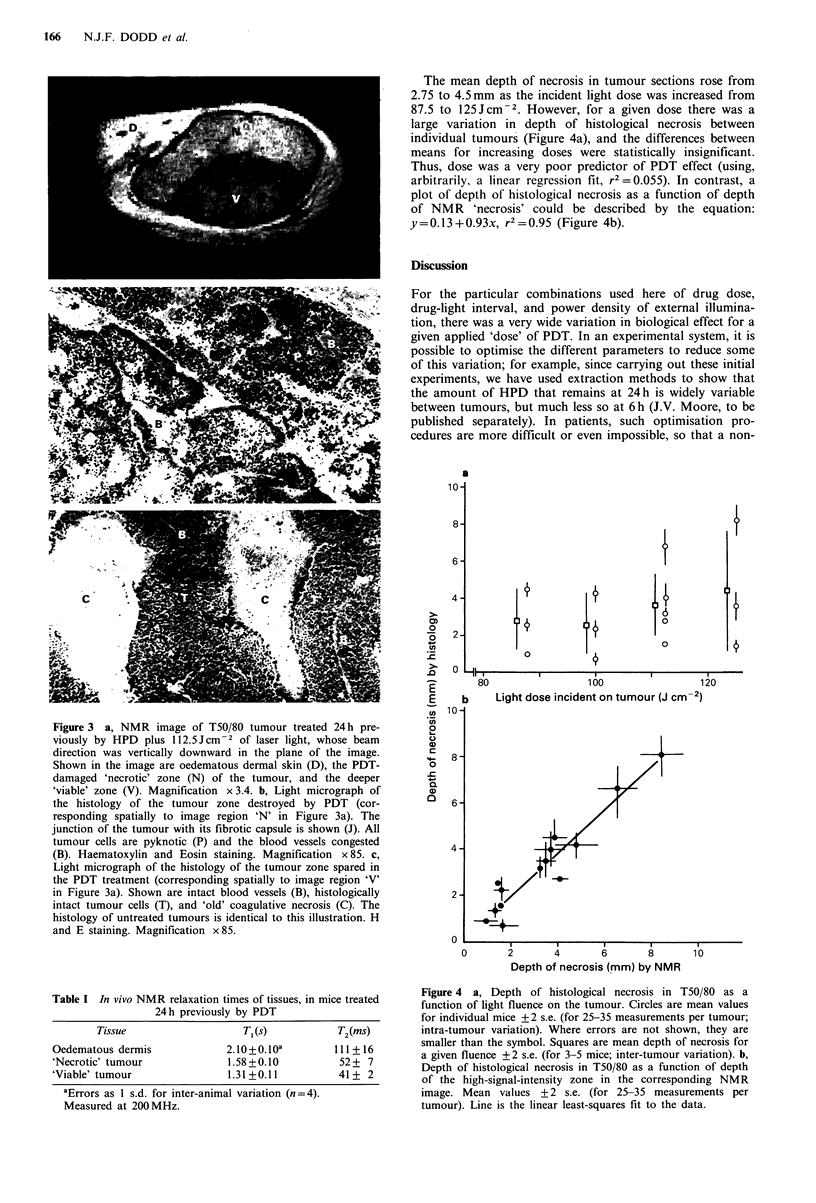

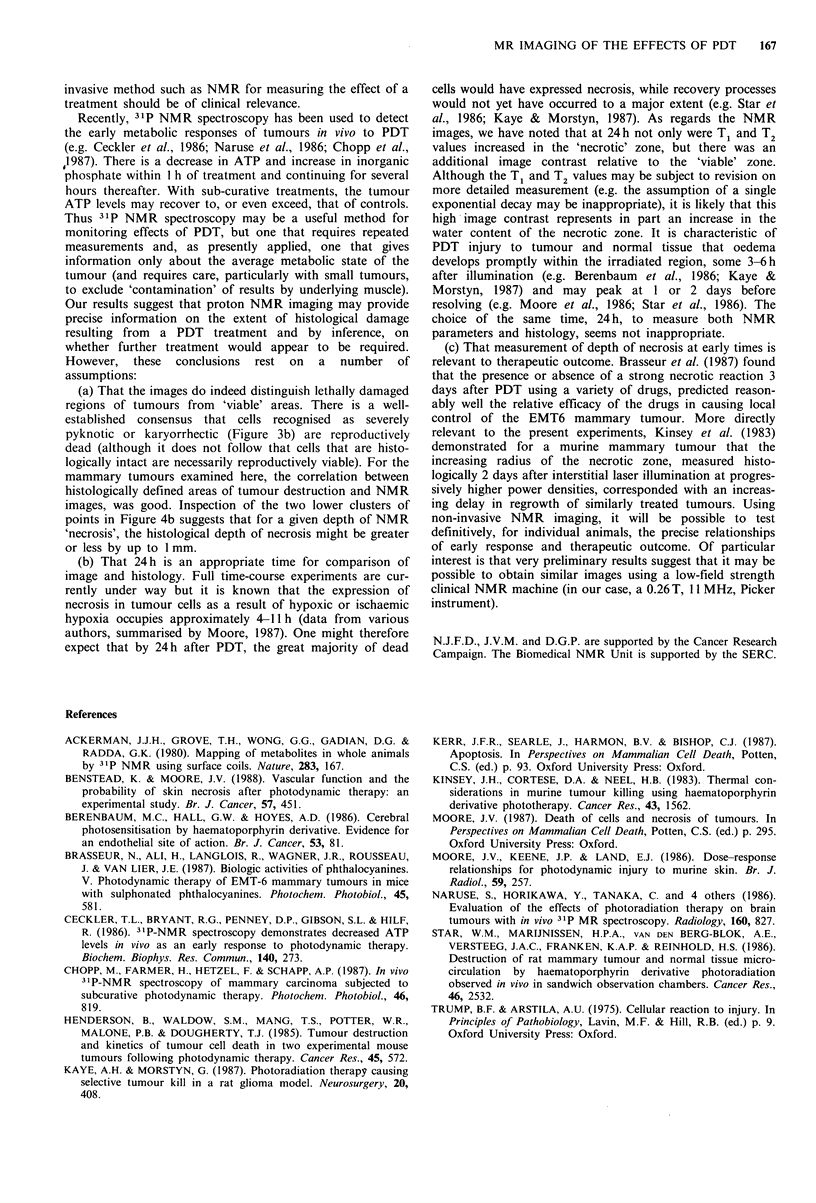


## References

[OCR_00473] Ackerman J. J., Grove T. H., Wong G. G., Gadian D. G., Radda G. K. (1980). Mapping of metabolites in whole animals by 31P NMR using surface coils.. Nature.

[OCR_00478] Benstead K., Moore J. V. (1988). Vascular function and the probability of skin necrosis after photodynamic therapy: an experimental study.. Br J Cancer.

[OCR_00483] Berenbaum M. C., Hall G. W., Hoyes A. D. (1986). Cerebral photosensitisation by haematoporphyrin derivative. Evidence for an endothelial site of action.. Br J Cancer.

[OCR_00488] Brasseur N., Ali H., Langlois R., Wagner J. R., Rousseau J., van Lier J. E. (1987). Biological activities of phthalocyanines--V. Photodynamic therapy of EMT-6 mammary tumors in mice with sulfonated phthalocyanines.. Photochem Photobiol.

[OCR_00495] Ceckler T. L., Bryant R. G., Penney D. P., Gibson S. L., Hilf R. (1986). 31P-NMR spectroscopy demonstrates decreased ATP levels in vivo as an early response to photodynamic therapy.. Biochem Biophys Res Commun.

[OCR_00501] Chopp M., Farmer H., Hetzel F., Schaap A. P. (1987). In vivo 31P-NMR spectroscopy of mammary carcinoma subjected to subcurative photodynamic therapy.. Photochem Photobiol.

[OCR_00507] Henderson B. W., Waldow S. M., Mang T. S., Potter W. R., Malone P. B., Dougherty T. J. (1985). Tumor destruction and kinetics of tumor cell death in two experimental mouse tumors following photodynamic therapy.. Cancer Res.

[OCR_00512] Kaye A. H., Morstyn G. (1987). Photoradiation therapy causing selective tumor kill in a rat glioma model.. Neurosurgery.

[OCR_00522] Kinsey J. H., Cortese D. A., Neel H. B. (1983). Thermal considerations in murine tumor killing using hematoporphyrin derivative phototherapy.. Cancer Res.

[OCR_00532] Moore J. V., Keene J. P., Land E. J. (1986). Dose-response relationships for photodynamic injury to murine skin.. Br J Radiol.

[OCR_00537] Naruse S., Horikawa Y., Tanaka C., Higuchi T., Sekimoto H., Ueda S., Hirakawa K. (1986). Evaluation of the effects of photoradiation therapy on brain tumors with in vivo P-31 MR spectroscopy.. Radiology.

[OCR_00541] Star W. M., Marijnissen H. P., van den Berg-Blok A. E., Versteeg J. A., Franken K. A., Reinhold H. S. (1986). Destruction of rat mammary tumor and normal tissue microcirculation by hematoporphyrin derivative photoradiation observed in vivo in sandwich observation chambers.. Cancer Res.

